# Supplementary material for: Physical Activity and Self-Reported Metabolic Syndrome Risk Factors in the Aboriginal Population in Perth, Australia, Measured Using an Adaptation of the Global Physical Activity Questionnaire (GPAQ)
Source: Int J Environ Res Public Health. 2021 Jun 2;18(11):5969. doi: 10.3390/ijerph18115969 (PMC8199758; doi:10.3390/ijerph18115969)
Supplement: Supplementary file 1 [file ijerph-18-05969-s001.zip › ijerph-1183071-supplementary.pdf]

Determining the motivators and barriers to exercise in the Australian Indigenous community.

This questionnaire is part of a PhD which will involve 2 studies.

Study 1- Identifying barriers and motivators for Indigenous People to engage and persist in an exercise program

This study will collect information relating to an Aboriginal individuals' perception of the barriers and motivators to aerobic and anaerobic exercise. A survey questionnaire with a sample of stakeholders (potential participants) and key informants with a series of questions designed to elicit the:

- Factors that impact uptake and retention
- Potential sustainability

The questionnaire will include broad questions relating to health and physical activity in general then focus on thoughts and perceptions of specific exercise types and settings. A second questionnaire the Global Physical Activity Questionnaire (GPAQ) attached to the back of this questionnaire will allow comparison of the Noongar community to other international Indigenous and non-Indigenous communities. This cross-sectional questionnaire will target approximately a minimum of 60 male and female adults aged between 18 – 60. There is no maximum number of participants that will be allowed to complete the survey.

Study 2 - The Intervention

The more people that complete the survey the better; the information from this questionnaire will help to identify the most appropriate form of exercise for an Aboriginal exercise intervention which forms the second study of this PhD. If the results indicate that the Aboriginal community prefer a gym or even a dance-based intervention, then that will form the basis of the intervention. The study aims to have 40 male and female Indigenous participants aged between 18 – 50 complete the exercise intervention.

While this questionnaire has been designed to be completed individually, it can easily be delivered in group setting for those who may prefer to complete it that way. i.e having the questionnaire administrator reading the questions to the group.
